# Evaluation of Uterine Carcinosarcoma and Uterine Endometrial Carcinoma Using Magnetic Resonance Imaging Findings and Texture Features

**DOI:** 10.7759/cureus.55916

**Published:** 2024-03-10

**Authors:** Saki Tsuchihashi, Keita Nagawa, Hirokazu Shimizu, Kaiji Inoue, Yoshitaka Okada, Yasutaka Baba, Kosei Hasegawa, Masanori Yasuda, Eito Kozawa

**Affiliations:** 1 Department of Radiology, Saitama Medical University Hospital, Saitama, JPN; 2 Department of Radiology, Japanese Red Cross Ogawa Hospital, Saitama, JPN; 3 Department of Diagnostic Radiology, Saitama Medical University International Medical Center, Saitama, JPN; 4 Department of Gynecologic Oncology, Saitama Medical University International Medical Center, Saitama, JPN; 5 Department of Diagnostic Pathology, Saitama Medical University International Medical Center, Saitama, JPN

**Keywords:** combined model, texture analysis, mri, uterine endometrial carcinoma, uterine carcinosarcoma

## Abstract

Aim

This study aimed to evaluate the diagnostic feasibility of magnetic resonance imaging (MRI) findings and texture features (TFs) for differentiating uterine endometrial carcinoma from uterine carcinosarcoma.

Methods

This retrospective study included 102 patients who were histopathologically diagnosed after surgery with uterine endometrial carcinoma (n=68) or uterine carcinosarcoma (n=34) between January 2008 and December 2021. We assessed conventional MRI findings and measurements (cMRFMs) and TFs on T2-weighted images (T2WI) and apparent diffusion coefficient (ADC) map, as well as their combinations, in differentiating between uterine endometrial carcinoma and uterine carcinosarcoma. The least absolute shrinkage and selection operator (LASSO) was used to select three features with the highest absolute value of the LASSO regression coefficient for each model and construct a discriminative model. Binary logistic regression analysis was used to analyze the disease models and conduct receiver operating characteristic analyses on the cMRFMs, T2WI-TFs, ADC-TFs, and their combined model to compare the two diseases.

Results

A total of four models were constructed from each of the three selected features. The area under the curve (AUC) of the discriminative model using these features was 0.772, 0.878, 0.748, and 0.915 for the cMRFMs, T2WI-TFs, ADC-TFs, and a combined model of cMRFMs and TFs, respectively. The combined model showed a higher AUC than the other models, with a high diagnostic performance (AUC=0.915).

Conclusion

A combined model using cMRFMs and TFs might be helpful for the differential diagnosis of uterine endometrial carcinoma and uterine carcinosarcoma.

## Introduction

Uterine carcinosarcoma (UCS) is a highly aggressive gynecological malignancy with epithelial (carcinoma) and mesenchymal (sarcoma) components [1]. It has a poor prognosis, with a reported five-year survival rate of 31% [2] and a mortality rate of 16% [3]. Even in patients with early-stage UCS, approximately 10% of cases are associated with distant metastases [4], and thus, the prognosis of UCS is worse than that of uterine endometrial carcinoma (UEC) [5]. While the preoperative diagnosis of these two diseases is commonly achieved through cervicovaginal cytology and endometrial biopsy, there are instances where UCS may be misdiagnosed due to its localized nature, resulting in the detection of only the cancerous component [6]. Importantly, the life expectancy in patients with stage I to III UEC has been reported, with retroperitoneal lymph node dissection known to improve the five-year survival rate by approximately 15% [7]. Furthermore, recent studies have confirmed the efficacy of anti-HER2 therapy with trastuzumab deruxtecan, a novel drug therapy for patients with unresectable cases [8]. Therefore, it is crucial for radiologists to effectively communicate the presence of UCS to clinicians prior to surgery instead of misdiagnosing it as UEC.

Magnetic resonance imaging (MRI) is a useful method for providing important diagnostic information on uterus lesions, such as those found in UEC and UCS. Inhomogeneity on T2-weighted images (T2WI), predominant high signal intensity compared to uterine myometrium on T2WI, markedly hyper- and hypointensity on T2WI, hyperintensity on T1-weighted images (T1WI), and the ratio of the thickness of endometrium and shape of tumor are characteristic plain MRI features of UCS [9-12]. Furthermore, the presence of a larger contrast defect area on contrast-enhanced T1WI, early enhancement area, and degree of tumor enhancement are characteristic MRI features of UCS after gadolinium enhancement [13-15]. Although previous studies have reported numerous valuable MRI findings for these two tumors, there are limitations to the use of only qualitative assessments using MRI.

Texture analysis is a method of analyzing images that enables the measurement of image characteristics by assessing the distribution, intensity, and patterns of pixels on the surface [16]. Textural features obtained from tumors via texture analysis are known to reflect intra-tumor heterogeneity in various tumors, suggesting the possibility of going beyond visual assessment [17,18]. Texture analysis of MRI has been reported to be useful in differentiating between leiomyosarcoma and leiomyoma in uterine tumors [19]. Additionally, it may help in the differential diagnosis between UEC and UCS. This is a novel approach as, to our knowledge, there have been no published reports on the use of MRI-based texture analysis to distinguish between UEC and UCS.

The aim of this study was to evaluate conventional MRI findings and measurements (cMRFMs) and texture features (TFs) of UCS and UEC to identify the most favorable features for discriminating between the two diseases and to evaluate the combined diagnostic performance of cMRFMs and TFs.

## Materials and methods

Patient population

This retrospective study was approved by the relevant institutional review board, and informed consent was obtained in the form of an opt-out option on the website (approval number 20-178). All experiments were conducted in accordance with the relevant guidelines and regulations.

We searched the gynecological oncology database for consecutive patients with UEC and UCS between January 2008 and December 2021. Patients who had undergone hysterectomy and preoperative MRI at our institution were considered eligible.

**Figure 1:**
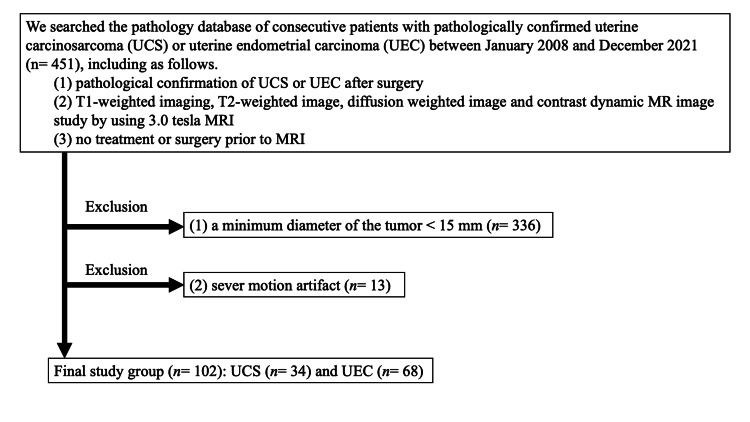
The flowchart shows the selection of the study population and exclusion criteria UCS - uterine carcinosarcoma; UEC - uterine endometrial carcinoma; MRI - magnetic resonance imaging

Patients who met the following inclusion criteria were eligible: 1) histopathological confirmed UEC or UCS after surgery, 2) 3.0-T MRI scan with T1WI, T2WI, diffusion-weighted image (DWI), apparent diffusion coefficient (ADC) maps, and dynamic contrast-enhanced fat-suppressed T1WI conducted at our hospital, and 3) no treatment or surgery prior to MRI. Therefore, a total of 451 consecutive patients were retrospectively reviewed. Patients with 1) tumors that were too small, with a minimum tumor diameter of less than 15 mm (n=336), and 2) severe motion artifacts (n=13) were excluded. Based on these criteria, finally, 102 patients (68 with UEC and 34 with UCS) were included. Clinical staging was based on the staging classification of the International Federation of Gynecology and Obstetrics (FIGO) 2008 [20]. The inclusion and exclusion criteria are summarized in Figure 1.

MRI protocol

Preoperative MRI was performed within two months (mean of 4±2.8 weeks, range of 1-9 weeks), using a 3.0-T system (Intera Achieva and Ingenia, Philips Healthcare, Best, The Netherlands). The MRI protocol included T2WI in the sagittal and axial planes, fat-suppressed T1WI in the sagittal plane, single-shot diffusion-weighted echo-planar imaging in the axial plane, and T1WI with dynamic contrast enhancement in the sagittal plane. Representative scan sequences and parameters are listed in Table 1. All patients received an intravenous injection of 20 mg butyl scopolamine (Buscopan; Boehringer Ingelheim, Ingelheim am Rhein, Germany) to relax the bowel wall and reduce peristaltic bowel movement before the MRI scan.

**Table 1:** Scan parameters of the magnetic resonance imaging T2WI - T2-weighted image; DWI - diffusion-weighted image; FsT1WI - fat-suppressed T1-weighted image; GdFsT1WI - gadolinium-enhanced fat-suppressed T1-weighted image; NEX - number of excitations

Sequence	T2WI	DWI	FsT1WI	GdFsT1WI
Repetition time (ms)	3000-5770	6000-12500	3.1-4.1	3.3-4.9
Echo time (ms)	90-100	70-78	1.6-1.8	1.6-1.8
Field of view (cm)	28×36	32×36	28×36	28×39
Matrix size	512×512	256×256	512×512	512×512
Slice thickness (mm)	4-5	4-5	4-5	2-3
Slice spacing (mm)	1	1	2	0
NEX	3	2	1	1
b value (s/mm^2^)	-	0, 500, 1000	-	-
Dynamic phase (s)	-	-	-	35, 65, 95, 125, 155

Conventional MRI findings and measurements

The evaluation of cMRFMs was performed independently by two radiologists with 25 and 30 years of experience in gynecological imaging who were blinded to patients' clinical and pathological data. All sections of the uterus and tumors on sagittal T2WI, sagittal fat-suppressed T1WI, and early and delayed phases of dynamic contrast-enhanced T1WI were evaluated.

The following 13 cMRFMs were recorded for UCS and UEC. (1) Solid components intensity on T2WI: zero represents homogeneous intensity, while one represents slightly heterogeneous and heterogeneous intensity [9-11]. (2) Predominant signal on T2WI: zero represents lower intensity compared to the outer myometrium, and one represents the same or higher intensity [9-11]. (3) Markedly hyperintense area on T2WI: one indicates the presence of regions within the solid component that demonstrate equal or higher intensity compared to urine, with areas exceeding a short axis 2 mm in size. The absence of such regions is indicated by zero [11]. (4) Markedly hypointense area on T2WI: one indicates the presence of an area within the solid component that exhibits the same or lower intensity and measures more than 2 mm in size, while zero indicates the absence of such an area [9-11]. (5) Extension to the uterine cervix with dilation of the cervical canal: one indicates the presence of extension to the uterine cervix with dilation of the cervical canal, while zero indicates its absence [11]. (6) Conspicuity of tumor margin: zero indicates a tumor margin with a prominently well-defined boundary on fat-saturated T1WI with gadolinium enhancement, while one indicates a tumor margin with a prominently ill-defined boundary. Specifically, zero represents a tumor margin where more than half is clearly delineated, whereas one represents a tumor margin where more than half appears ill-defined [10]. (7) Hyperintense areas on T1WI: one indicates the presence of regions with a short axis exceeding 2 mm and exhibiting signal intensity like that of fat, while a score of zero indicates the absence of such regions [10]. (8) Early enhancement area: one indicates the presence of early enhancement areas with a short axis exceeding 2 mm on the early phase of fat-saturated T1WI with gadolinium enhancement compared to the uterine outer myometrium enhancement, while a score of zero indicates the absence of such an area [10]. (9) The percentage of contrast defect area volume on the delay phase: zero indicates 0-25% defect areas within solid component, and one indicates 26-100% [9-11]. (10) Degree of tumor enhancement: zero indicates lower intensity compared to the uterine outer myometrium on the delay phase, while one indicates almost the same or higher intensity [9-12]. (11) Ascites: zero means absent, and one means present. (12) The tumor maximum diameter: the maximum diameter is measured on delayed phases of fat-saturated T1WI with gadolinium enhancement. (13) Maximum anteroposterior dimensions of the uterus (AT) and the thickness of the endometrium (ET) ratio (ET/AT ratio) were measured as previously described [12]. The cMRFMs from (1) to (11) were evaluated using all slices of each case. The cMRFMs data assessed were used for inter-reader reproducibility tests. In qualitative analysis evaluation, interobserver discrepancies were resolved by consensus.

MRI texture features

**Figure 2:**
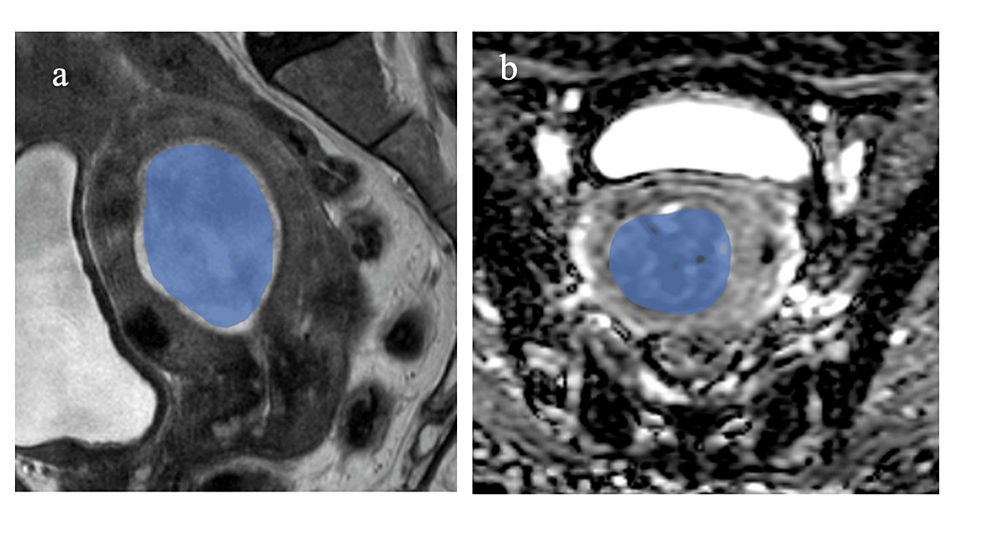
Segmentation in a 75-year-old woman with uterine carcinosarcoma (a) Sagittal T2-weighted image and (b) axial apparent diffusion coefficient map. The maximum tumor slice of each sagittal or axial T2-weighted image and axial apparent diffusion coefficient map is selected. An irregular two-dimensional region of interest is drawn manually to contain the outline borders of the entire region of the largest cross-sectional tumors using open-source software (ITK-SNAP version 3.8.0).

We obtained TFs derived from T2WI-TFs and ADC-TFs. Segmentation was performed using the open-source software (ITK-SNAP version 3.8.0). In each case, a slice of the sagittal and axial T2WI and ADC map containing the maximum section of the tumor was selected. A radiologist with 30 years of experience chose the most appropriate slice, and then two radiologists with 25 and 30 years of experience in gynecological imaging were asked to draw on the identified slice. An irregular two-dimensional region of interest (ROI) was referenced using contrast-enhanced images to accurately determine the precise location of the tumor. Tumor boundaries were defined to include the solid component of the tumor and exclude the border region to decrease partial volume effects (Figure 2). All radiologists were blinded to the clinical and pathological information. The TFs were extracted using an open-source Python package (PyRadiomics version 2.1.0; www.radiomics.io/pyradiomics.html). The extracted TFs included 18 first-order statistics, 24 gray-level co-occurrence matrices (GLCM), 16 gray-level run-length matrices (GLRLM), 16 gray-level size zone matrices (GLSZM), 14 gray-level dependence matrices, and five neighborhood gray-level difference matrix features.

Statistical analysis

Conventional MRI findings and measurement data were analyzed for the total number of cases in our dataset. Quantitative data regarding the mean and standard deviation of the MRI TFs were also calculated. Interobserver agreement was calculated using the kappa statistic and interclass correlation coefficients (ICCs). Kappa values and ICCs scores were interpreted according to the following criteria: zero as poor, 0.01-0.20 as minor, 0.21-0.40 as normal, 0.41-0.60 as moderate, 0.61-0.80 as substantial, and 0.81-1.00 as excellent [21,22]. We used kappa values for the categorical data of conventional MRI findings and ICC scores for the quantitative data of measurements and TFs [17]. In univariate analyses, the χ2 test was used for qualitative data and the Mann-Whitney U test for quantitative data. A significance level of p<0.05 was adopted.

Classification model development

We constructed a cMRFMs model, a T2WI-TFs model, an ADC-TFs model, and a combined model using all selected cMRFMs and TFs. Prior to conducting consecutive statistical analyses, all numeric values were Z-standardized. This standardization aimed to minimize the effects associated with variations between two MRI machines, as well as differences in scales ranging from small values like 0.01 to larger ones like 1000. To construct discriminant models for distinguishing between UEC and UCS, we performed dimensional reduction of the obtained features. First, we reduced the number of features using interobserver reproducibility tests (kappa and ICC values ≥0.6) and univariate analysis (p<0.05). Additionally, given the large number of features, we adopted the least absolute shrinkage and selection operator (LASSO) algorithm using tenfold cross-validation, which is a suitable method for the regression of high-dimensional data. We excluded features with high collinearity (r≥0.7 with Pearson's correlation) from the analysis. To avoid overfitting, we selected three features with the highest absolute values of the LASSO regression coefficient for each model. To address the issue of overfitting caused by the small sample size, we employed ten-fold cross-validation and repeated the process 10 times to build each model. Four models were analyzed using binary logistic regression analysis. The area under the curve (AUC) of the receiver operating characteristic analysis was performed on the independent discriminative features, and the differences in the AUC between the discriminative models were compared using the DeLong method with Bonferroni correction. Statistical significance was set at p<0.05/4=0.0125. Accuracy, sensitivity, specificity, and AUC were derived from the classification results. Statistical analyses were performed using JMP Pro (v16, SAS Institute, Cary, NC, USA) and SPSS version 29 (IBM Corp., Armonk, NY, USA). The analyses were performed independently by two radiologists, and agreement between readers was assessed to ensure consistency between readers.

## Results

Clinical characteristics

Overall, 102 patients, including 34 with UCS and 68 with UEC, were evaluated. The clinical characteristics of the patients are shown in Table 2. No significant difference was found in the patient's age and 2008 FIGO stage [20] between the UCS and UEC groups. In the UCS group, 13 (38 %) patients were homologous, and 21 (62%) were heterologous. In the UEC group, the distribution of grade one, grade two, grade three, serous carcinoma, and undifferentiated carcinoma was as follows: 30 (44%), 18 (26%), 17 (25%), two (3%), and one (2%), respectively.

**Table 2:** Clinical characteristics of the study patients UCS - uterine carcinosarcoma; UEC - uterine endometrial carcinoma

Clinical characteristics	UCS (n=34)	UEC (n=68)	p-value
Age (years), mean (range±SD)	67 (39-88±11)	66 (39-97±13)	0.69
Clinical stage, n (%)			
ⅠA	12 (35%)	23 (34%)	-
ⅠB	12 (35%)	19 (28%)	-
Ⅱ	1 (3%)	5 (7%)	-
ⅢA	2 (6%)	3 (4%)	-
ⅢB	0 (0%)	0 (0%)	-
ⅢC1	0 (0%)	6 (9%)	-
ⅢC2	2 (6%)	4 (6%)	-
ⅣA	0 (0%)	1 (2%)	-
ⅣB	5 (15%)	7 (10%)	-
UCS type			
Homologous types, n (%)	13 (38%)	-	-
Heterologous types, n (%)	21 (62%)	-	-
UEC grade			
Grade 1, n (%)	-	30 (44%)	-
Grade 2, n (%)	-	18 (26%)	-
Grade 3, n (%)	-	17 (25%)	-
Serous adenocarcinoma, n (%)	-	2 (3%)	-
Undifferentiated carcinoma, n (%)	-	1 (2%)	-

Conventional MRI features assessment

**Figure 3:**
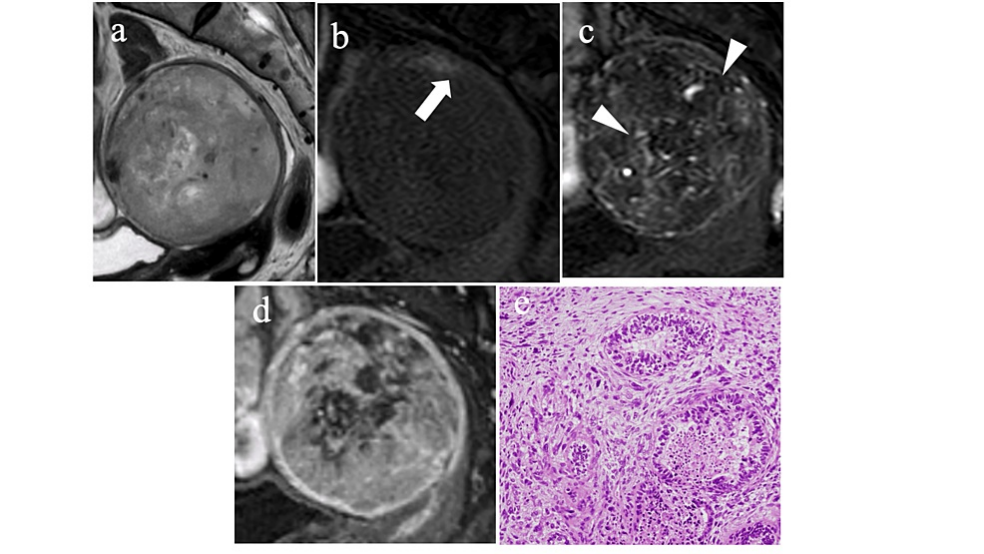
A 76-year-old woman with uterine carcinosarcoma (a) Mass in the endometrial cavity (6×6 cm), heterogeneous and hyperintense signals compared to myometrium on sagittal T2-weighted image. (b) An area of hyperintense signal with a minimum diameter of 2 mm in the patient with uterine carcinosarcoma on sagittal fat-suppressed T1WI (see arrow). (c) In the early phase of sagittal gadolinium-enhanced fat-suppressed contrast-enhanced T1WI, high contrast effect areas are present compared to the myometrium (see head). (d) In the delayed phase, more than 26% of contrast defect areas are present in the endometrial cavity. The degree of tumor enhancement is intermediate compared to the myometrium. (e) Photomicrograph of representative histologic section (hematoxylin-eosin stain; original magnification, ×20) demonstrates both adenocarcinomatous components and sarcomatous elements. T1W1 - T1-weighted image

**Figure 4:**
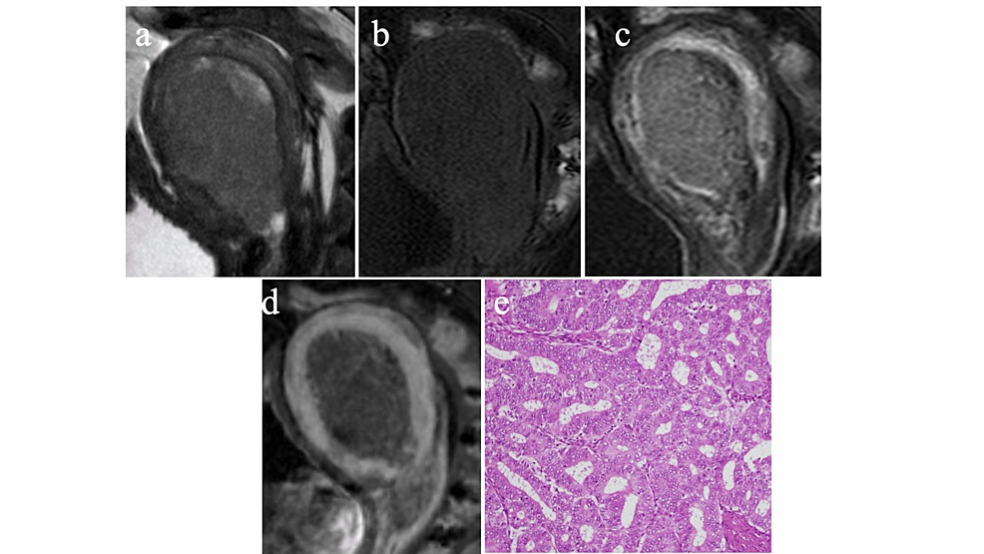
A 71-year-old woman with uterine endometrioid carcinoma (grade 1) (a) The mass in the endometrial cavity is approximately 6×3 cm; the homogeneous and hypointense signal on the sagittal T2-weighted image. (b) There is no hyperintense area on the fat-suppressed sagittal T1WI. (c) The area of contrast enhancement is unclear on fat-suppressed T1WI. (d) The contrast defect area is 0% on the delayed phase, and the degree of tumor enhancement is low. (e) Photomicrograph of representative histologic section (hematoxylin-eosin stain; original magnification, ×20) demonstrates predominantly large-sized fused glandular architectures. T1W1 - T1-weighted image

We compared cMRFMs between the groups, as shown in Table 3. Representative cases of both disease types are shown in Figures 3 and 4. In the univariate analysis, the eight variables of solid component intensity on T2WI, predominant signal intensity on T2WI, markedly hyperintense area on T2WI, markedly hypointense area on T2WI, hyperintense area on T1WI, early enhancement area, percentage of the volume of the contrast defect area, and degree of tumor enhancement were identified as significant differentiation factors for the two disease groups (p<0.05). No significant difference was observed in the conspicuity of the tumor margin, extension to the uterine cervix with dilation of the cervical canal, ascites, tumor maximum diameter, and ET/AP ratio. We used kappa and ICC values to examine interobserver variability and found excellent and substantial or excellent agreement for nine of all 13 cMRFMs.

**Table 3:** Comparison of conventional MRI findings and measurements in study patients UCS - uterine carcinosarcoma; UEC - uterine endometrial carcinoma; ICC - intraclass correlation coefficient; T2WI - T2-weighted image; T1WI - T1-weighted image; ET/AP ratio - the ratio of the thickness of the endometrium to the largest anteroposterior dimensions of the uterus

Conventional MRI findings	UCS (n=34)		UEC (n=68)		p-value	Kappa statics/ICC
	0	1	0	1		
Solid components intensity on T2WI	15	19	55	13	<0.001	0.755
Predominant signal intensity on T2WI	21	13	63	5	<0.001	0.52
Markedly hyperintense area on T2WI	10	24	42	26	0.003	0.783
Markedly hypointense area on T2WI	5	29	40	28	<0.001	0.455
Extension to the uterine cervix with dilation of the cervical canal	28	6	49	19	0.332	0.873
Conspicuity of tumor margin	6	28	20	48	0.613	0.023
Hyperintense area on T1WI	14	20	55	13	<0.001	0.79
Early enhancement area	10	24	55	13	<0.001	0.768
Percentage of the volume of the contrast defect area	21	13	64	4	<0.001	0.752
Degree of tumor enhancement	18	16	62	6	<0.001	0.676
Ascites	13	21	25	43	0.824	0.199
Measurements						
Tumor maximum diameter (mm), mean (range±SD)	66 (25-136±28)		58 (19-161±25)		0.14	0.852
ET/AP ratio, mean (range±SD)	0.74 (0.51-0.93±0.12)		0.67 (0.29-0.95±0.15 )		0.195	0.645

Texture features assessment

The mean ROI sizes determined by the two radiologists were 1098 and 919 mm^2^ for UCS and 516 and 464 mm^2 ^for UEC, respectively. Among 93 TFs, the mean ICC value was 0.856±0.139 and 0.877±0.083 for T2WI-TFs and ADC-TFs, and the numbers of features with excellent ICC (≥0.8) were 70 and 84, respectively. The Z-standardized representative TFs are shown in Table 4. Using univariate analysis, on the first-order features, 90^th^ percentile, median, and mean ADC-TFs for UCS were significantly higher than those for UEC. Using p<0.05 in univariate analysis of the Mann-Whitney U test, the number of features was further reduced to 61 and 75, respectively, as shown in Table 5.

**Table 4:** Z-standardized representative texture features from the least absolute shrinkage and selection operator regression analysis on T2-weighted image and apparent diffusion coefficient map UCS - uterine carcinosarcoma; UEC - uterine endometrial carcinoma; ICC - intraclass correlation coefficient; GLCM - gray-level co-occurrence matrix; GMSZM - gray-level size zone matrix

Parameters	mean±SD		p-value	ICC
	UCS	UEC		
Texture features on T2-weighted image				
First-order 10^th^ percentile	0.4898±0.9863	-0.2449±0.9196	0.001	0.854
First-order energy	0.4502±1.5002	-0.2251±0.4946	0.001	0.9386
First-order mean	0.7184±1.0229	-0.3592±0.7741	<0.0001	0.9495
First-order median	0.7319±1.0064	-0.3656±0.7751	<0.0001	0.95
GLCM correlation	0.5373±0.9487	-0.2686±0.9190	<0.0001	0.8921
GLCM sum average	0.8886±1.0749	-0.4443±0.5808	<0.0001	0.8611
Texture features on the apparent diffusion coefficient				
First-order 90^th^ percentile	0.4747±1.2231	-0.2371±0.7693	0.0013	0.872
First-order total energy	0.6072±1.3762	-0.3036±0.5422	<0.0001	0.9364
First-order mean	0.3711±1.2549	-0.1856±0.7918	0.0163	0.9472
First-order median	0.3437±1.2609	-0.1719±0.7966	0.0229	0.9658
GLCM maximum probability	-0.4628±0.8549	0.2314±0.9923	0.0001	0.9404
GLSZM high gray-level zone emphasis	0.6657±1.3649	-0.3328±0.5026	0.0002	0.823

**Table 5:** Z-standardized texture features of T2-weighted image and apparent diffusion coefficient map UCS - uterine carcinosarcoma; UEC - uterine endometrial carcinoma; GLCM - gray-level co-occurrence matrix; GLSZM - gray-level size zone matrix; GLRLM - gray-level run-length matrix; GLDM - gray-level dependence matrix; NGTDM - neighboring gray-tone difference matrix; Imc -  informational measure of correlation

Texture features	mean ± standard deviation		p-value
	UCS	UEC	
Z-standardized texture features on T2-weighted images			
First-order			
10^th^ percentile	0.4898 ± 0.9863	-0.2449 ± 0.9196	0.0010
90^th^ percentile	0.7787 ± 1.1318	-0.3893 ± 0.6442	<0.0001
Energy	0.4502 ± 1.5002	-0.2251 ± 0.4946	0.0010
Entropy	0.7538 ± 1.0503	-0.3769 ± 0.7291	<0.0001
Interquartile range	0.6570 ± 1.4273	-0.328481 ± 0.4189	<0.0001
Maximum	0.7321 ± 1.0063	-0.3661 ± 0.7345	<0.0001
Mean absolute deviation	0.7060 ± 1.3262	-0.3530 ± 0.5116	<0.0001
Mean	0.7184 ± 1.0229	-0.3592 ± 0.7741	<0.0001
Median	0.7319 ± 1.0064	-0.3656 ± 0.7751	<0.0001
Range	0.7256 ± 1.0449	-0.3638 ± 0.7542	<0.0001
Robust mean absolute deviation	0.6700 ± 1.4067	-0.3350 ± 0.4372	<0.0001
Root mean squared	0.7260 ± 1.0312	-0.3630 ± 0.7632	<0.0001
Total energy	0.5050 ± 1.2841	-0.2525 ± 0.7079	<0.0001
Uniformity	-0.6878 ± 0.8309	0.3439 ± 0.8986	<0.0001
Variance	0.6352 ± 1.4748	-0.3177 ± 0.3593	<0.0001
GLCM			
Autocorrelation	0.8389 ± 1.2811	-0.4195 ± 0.4042	<0.0001
Cluster prominence	0.5085 ± 1.5770	-0.2542 ± 0.2928	<0.0001
Cluster tendency	0.6327 ± 1.4789	-0.3163 ± 0.0354	<0.0001
Contrast	0.5216 ± 1.2478	-0.2608 ± 0.7304	0.0003
Correlation	0.5373 ± 0.9487	-0.2686 ± 0.9190	<0.0001
Difference average	0.4932 ± 1.1424	-0.2466 ± 0.8243	<0.0001
Difference entropy	0.5150 ± 1.0802	-0.2575 ± 0.8549	0.0008
Difference variance	0.5280 ± 1.2510	-0.2640 ± 0.7242	0.0003
Inverse difference	-0.4009 ± 1.0557	0.2005 ± 0.9144	0.0045
Inverse difference moment	-0.3908 ± 1.0428	0.1954 ± 0.9250	<0.0001
Imc 1	-0.6060 ± 1.0832	0.3030 ± 0.8062	<0.0001
Imc 2	0.5377 ± 0.8549	-0.2689 ± 0.9630	<0.0001
Inverse variance	-0.4609 ± 1.0325	0.2304 ± 0.9059	0.0011
Joint average	0.8886 ± 1.0749	-0.4443 ± 0.5808	<0.0001
Joint energy	-0.6009 ± 0.6890	0.3005 ± 0.9993	<0.0001
Joint entropy	0.7103 ± 1.0155	-0.3551 ± 0.7846	<0.0001
Maximal correlation coefficient	0.6376 ± 0.7349	-0.3188 ± 0.9654	<0.0001
Maximum probability	-0.5604 ± 0.6576	0.2802 ± 1.0273	<0.0001
Sum average	0.8886 ± 1.0749	-0.4443 ± 0.5808	<0.0001
Sum entropy	0.7405 ± 1.4671	-0.3702 ± 0.7419	<0.0001
Sum squares	0.6337 ± 1.4745	-0.3169 ± 0.3618	<0.0001
GLDM			
Dependence entropy	0.7632 ± 1.0806	-0.3816 ± 0.6993	<0.0001
Dependence non-uniformity	0.4008 ± 1.3587	-0.2004 ± 0.6899	0.0032
Dependence non-uniformity normalized	0.3199 ± 1.1088	-0.1600 ± 0.9078	0.0359
Gray-level variance	0.6352 ± 1.4750	-0.3176 ± 0.3589	<0.0001
High gray-level emphasis	0.8396 ± 1.2791	-0.4198 ± 0.4064	<0.0001
Large dependence emphasis	-0.2792 ± 1.0264	0.1396 ± 0.9640	0.0347
Large dependence high gray-level emphasis	0.8186 ± 1.2962	-0.4093 ± 0.4121	<0.0001
Large dependence low gray-level emphasis	-0.5481 ± 0.3314	0.2741 ± 1.1066	<0.0001
Small dependence emphasis	0.3791 ± 1.0852	0.3141 ± 1.0662	0.0105
Small dependence high gray-level emphasis	0.7960 ± 1.3250	-0.3980 ± 0.4004	<0.0001
Small dependence low gray-level emphasis	-0.6455 ± 0.4347	0.3227 ± 1.0475	<0.0001
GLRLM			
Gray-level non-uniformity normalized	-0.6940 ± 0.8400	0.3470 ± 0.8907	<0.0001
Gray-level variance	0.6390 ± 1.4616	-0.3195 ± 0.3801	<0.0001
High gray-level run emphasis	0.8398 ± 1.2752	-0.4199 ± 0.4119	<0.0001
Long run emphasis	-0.2712 ± 1.0319	0.1356 ± 0.9628	0.0318
Long run high gray-level emphasis	0.8444 ± 1.2712	-0.4222 ± 0.4108	<0.0001
Long run low gray-level emphasis	-0.6248 ± 0.3812	0.2918 ± 1.0898	<0.0001
Low gray-level run emphasis	0.7642 ± 1.0753	0.3124 ± 1.0671	<0.0001
Run entropy	0.7642 ± 1.7053	-0.3821 ± 0.7024	<0.0001
Run length non-uniformity	0.3969 ± 1.3652	-0.1985 ± 0.6853	0.0037
Run length non-uniformity normalized	0.3467 ± 1.0386	-0.1733 ± 0.9406	0.0150
Run percentage	0.3155 ± 1.0371	-0.1578 ± 0.9497	0.0150
Run variance	-0.2318 ± 1.0610	0.1159 ± 0.9550	0.0183
Short run emphasis	0.3376 ± 1.0230	-0.1688 ± 0.9515	0.0160
Short run high gray-level emphasis	0.8350 ± 1.2805	-0.4175 ± 0.4114	<0.0001
Short run low gray-level emphasis	-0.6322 ± 0.3922	0.3161 ± 1.0619	<0.0001
GLSZM			
Gray-level non-uniformity	0.1445 ± 1.0286	-0.0723 ± 0.9851	<0.0001
Gray-level non-uniformity normalized	-0.7103 ± 0.8705	0.3552 ± 0.8661	<0.0001
Gray-level variance	0.6492 ± 1.4132	-0.3246 ± 0.4505	<0.0001
High gray-level zone emphasis	0.8386 ± 1.2632	-0.4193 ± 0.4315	<0.0001
Large area emphasis	-0.2538 ± 0.6726	0.1269 ± 1.1116	0.0296
Large area high gray-level emphasis	0.6645 ± 1.3585	-0.3222 ± 0.5123	<0.0001
Large area low gray-level emphasis	-0.3313 ± 0.1817	0.1656 ± 1.18644	<0.0001
Size zone non-uniformity	0.4507 ± 1.2586	-0.2253 ± 0.7567	<0.0001
Size zone non-uniformity normalized	0.3974 ± 1.1326	-0.1987 ± 0.8691	0.0008
Low gray-level zone emphasis	-0.6087 ± 0.3886	0.3043 ± 1.0728	0.0034
Small area emphasis	0.3773 ± 1.1350	-0.1887 ± 0.8744	0.0032
Small area high gray-level emphasis	0.8170 ± 1.2833	-0.4085 ± 0.4339	<0.0001
Small area low gray-level emphasis	-0.6153 ± 0.4065	0.3077 ± 1.0667	<0.0001
Zone entropy	0.6936 ± 1.1050	-0.3468 ± 0.7348	<0.0001
Zone percentage	0.3691 ± 1.051	-0.1846 ± 0.9272	0.0107
NGTDM			
Busyness	-0.5605 ± 0.7436	0.2803 ± 0.9980	<0.0001
Complexity	0.6516 ± 1.2036	-0.3258 ± 0.6861	<0.0001
Strength	0.4584 ± 1.4504	-0.2292 ± 0.5580	<0.0001
Z-standardized texture features on apparent diffusion coefficient maps			
First-order			
90^th^ percentile	0.4747 ± 1.2231	-0.2371 ± 0.7693	0.0013
Energy	0.5758 ± 1.3960	-0.2874 ± 0.5443	<0.0001
Entropy	0.5881 ± 1.0869	-0.2941 ± 0.8139	<0.0001
Mean	0.3711 ± 1.2549	-0.1856 ± 0.7918	0.0163
Median	0.3437 ± 1.2609	-0.1719 ± 0.7966	0.0229
Minimum	-0.3315 ± 1.1164	0.1658 ± 0.9000	0.0201
Root mean squared	0.3822 ± 1.2552	-0.1911 ± 0.7876	0.0134
Total energy	0.6072 ± 1.3762	-0.3036 ± 0.5422	<0.0001
Uniformity	-0.5031 ± 0.9577	0.2515 ± 0.9290	<0.0001
GLCM			
Autocorrelation	0.6670 ± 1.3814	-0.3335 ± 0.4785	0.0003
Contrast	0.4750 ± 1.2281	-0.2375 ± 0.7770	0.0007
Difference average	0.5204 ± 1.1795	-0.2602 ± 0.7916	0.0006
Difference entropy	0.5310 ± 1.6179	-0.2655 ± 0.8589	0.0005
Inverse difference	-0.5064 ± 1.004	0.2532 ± 0.9035	0.0006
Inverse difference moment	-0.4967 ± 0.9799	0.2483 ± 0.9202	0.0007
Inverse variance	-0.4938 ± 1.0560	0.2469 ± 0.8790	0.0006
Joint average	0.6472 ± 1.2680	-0.3236 ± 0.6298	0.0003
Joint energy	-0.4534 ± 0.8490	0.2276 ± 0.9980	<0.0001
Joint entropy	0.6222 ± 1.1013	-0.3111 ± 0.7844	<0.0001
Maximum probability	-0.4628 ± 0.8549	0.2314 ± 0.9923	0.0001
Sum average	0.6472 ± 1.2681	-0.3236 ± 0.6298	0.0003
Sum entropy	0.5853 ± 1.0805	-0.2927 ± 0.8196	<0.0001
GLDM			
Dependence entropy	0.5736 ± 1.1005	-0.2868 ± 0.8127	<0.0001
Dependence non-uniformity	0.5831 ± 1.4244	-0.2916 ± 0.4992	<0.0001
Dependence non-uniformity normalized	0.4827 ± 1.1224	-0.2414 ± 0.8424	0.0012
Dependence variance	-0.3980 ± 0.9273	0.1990 ± 0.9815	0.0045
Gray-level non-uniformity	0.2259 ± 0.9946	-0.1129 ± 0.9907	0.0197
High gray-level emphasis	0.6691 ± 1.3812	-0.3345 ± 0.4765	0.0002
Large dependence emphasis	-0.4291 ± 0.8458	0.2146 ± 1.0075	0.0016
Large dependence high gray-level emphasis	0.5687 ± 1.2828	-0.2843 ± 0.6714	0.0011
Large dependence low gray-level emphasis	-0.2973 ± 0.6363	0.1482 ± 1.1141	0.0062
Low gray-level emphasis	-0.4371 ± 0.6103	0.2186 ± 1.0856	0.004
Small dependence emphasis	0.4606 ± 1.0232	-0.2303 ± 0.9112	0.0016
Small dependence high gray-level emphasis	0.6603 ± 1.4038	-0.3302 ± 0.4528	0.0020
Small dependence low gray-level emphasis	-0.4675 ± 0.7728	0.2338 ± 1.0232	<0.0001
GLRLM			
Gray-level non-uniformity	0.2967 ± 1.0484	-0.1483 ± 0.9482	0.0080
Gray-level non-uniformity normalized	-0.5104 ± 0.9636	0.2552 ± 0.9230	0.0002
High gray-level run emphasis	0.6687 ± 1.3771	-0.3443 ± 0.4829	0.0002
Long run emphasis	-0.4155 ± 0.8065	0.2077 ± 1.0275	0.0024
Long run high gray-level emphasis	0.6495 ± 1.3525	-0.3248 ± 0.5341	0.0050
Long run low gray-level emphasis	-0.3955 ± 0.5764	0.1978 ± 1.1067	0.001
Low gray-level run emphasis	-0.4604 ± 0.6176	0.1978 ± 1.1067	0.0001
Run length non-uniformity	0.5723 ± 1.3588	0.2320 ± 1.0762	<0.0001
Run length non-uniformity normalized	0.4697 ± 0.9412	-0.2862 ± 0.5906	0.0011
Run percentage	0.4503 ± 0.9049	-0.2348 ± 0.9504	0.0015
Run variance	-0.3960 ± 0.7958	-0.2251 ± 0.9746	0.0030
Short run emphasis	0.4593 ± 0.8957	-0.2297 ± 0.9755	0.0011
Short run high gray-level emphasis	0.6699 ± 1.3808	-0.3350 ± 0.4762	0.0020
Short run low gray-level emphasis	-0.4753 ± 0.6594	0.2376 ± 1.0617	<0.0001
GLSZM			
Gray-level non-uniformity	0.4603 ± 1.2054	-0.2302 ± 0.7940	<0.0001
Gray-level non-uniformity normalized	-0.5196 ± 0.9797	0.2598 ± 0.9106	<0.0001
Gray-level variance	0.4184 ± 1.1518	-0.2092 ± 0.8490	0.0002
High gray-level zone emphasis	0.6657 ± 1.3649	-0.3328 ± 0.5026	0.0002
Large area emphasis	-0.3530 ± 0.6074	0.1765 ± 1.1095	0.0027
Large area high gray-level emphasis	0.2472 ± 0.8881	-0.1236 ± 1.0356	0.0016
Large area low gray-level emphasis	-0.3304 ± 0.4687	0.1655 ± 1.1473	<0.0001
Size zone non-uniformity	0.5818 ± 1.4626	0.2426 ± 1.0447	<0.0001
Size zone non-uniformity normalized	0.4427 ± 1.0403	-0.2909 ± 0.4430	0.0023
Low gray-level zone emphasis	-0.4852 ± 0.6935	-0.2213 ± 0.9084	<0.0001
Small area emphasis	0.4341 ± 0.9855	-0.2171 ± 0.9411	0.0024
Small area high gray-level emphasis	0.6617 ± 1.3795	-0.3308 ± 0.4868	0.0002
Small area low gray-level emphasis	-0.4743 ± 0.8356	0.2372 ± 0.9961	< 0.0001
Zone entropy	0.5421 ± 1.0281	-0.2710 ± 0.8736	0.0001
Zone percentage	0.4516 ± 0.9805	-0.2258 ± 0.9374	0.0021
Zone variance	-0.3319 ± 0.5971	0.1660 ± 1.1171	0.0038
NGTDM			
Busyness	-0.1388 ± 0.9355	0.0694 ± 1.0304	< 0.0001
Coarseness	-0.4377 ± 0.9288	0.2188 ± 0.9679	0.0004
Complexity	0.5409 ± 1.3805	-0.2705 ± 0.5883	0.0251
Contrast	0.2316	-0.1158 ± 0.9930	< 0.0001

Classification model development assessment

We evaluated the performance of the diagnostic model using the best parameters for cMRFMs, T2WI-TFs, ADC-TFs, and combined models. For the discriminative model construction, we selected three features based on their higher absolute values of the LASSO regression coefficient. In the cMRFMs model, we obtained three features: the markedly hypointense area on T2WI, the early enhancement area, and the percentage of the contrast defect area. From the T2WI-TFs model, we selected first-order median, GLCM correlation, and GLCM sum average. For the ADC-TFs model, we chose first-order total energy, GLCM maximum probability, and GLSZM high gray-level zone emphasis. In addition, for the combined model of cMRFMs and TFs, we selected the first-order median on T2WI-TFs, GLCM sum average on T2WI-TFs, and early enhancement area on cMRFMs.

The cMRFMs model achieved an AUC of 0.772, accuracy of 0.643, sensitivity of 0.870, and specificity of 0.416. The T2WI-TFs model had an AUC of 0.878, accuracy of 0.831, sensitivity of 0.943, and specificity of 0.631. The ADC-TFs model achieved an AUC of 0.748, accuracy of 0.750, sensitivity of 0.945, and specificity of 0.365. Finally, the combined model had an AUC of 0.915, accuracy of 0.851, sensitivity of 0.946, and specificity of 0.655 (Table 6). The combined model showed the highest AUC compared to the other models. The diagnostic performance of the combined model was significantly higher than that of the cMRFMs model (p<0.001), T2WI-TFs model (p<0.001), and ADC-TFs model (p<0.001).

**Table 6:** Diagnostic performance of the models for differentiating uterine carcinosarcoma and uterine endometrial carcinoma cMRFMs - conventional MRI findings and measurements; AUC - area under curve; T2WI - T2-weighted image; T1WI - T1-weighted image; TFs - texture features; GLCM - gray-level co-occurrence matrix; ADC - apparent diffusion coefficient; GLSZM - gray-level size zone matrix

Model	Feature	Accuracy	Sensitivity	Specificity	AUC
cMRFMs model	Markedly hypointense area on T2WI	0.643	0.870	0.416	0.772
	Early enhancement area				
	Percentage of the volume of the contrast defect area				
T2WI-TFs model	First-order median	0.831	0.943	0.631	0.878
	GLCM correlation				
	GLCM sum average				
ADC-TFs model	First- order total energy	0.750	0.945	0.365	0.748
	GLCM maximum probability				
	GLSZM high gray-level zone emphasis				
Combined model of cMRFMs and TFs	First-order median on T2WI TFs	0.851	0.946	0.655	0.915
	GLCM sum average on T2WI TFs				
	Early enhancement area				

## Discussion

In this study investigating the performance of conventional MRI findings and quantitative features from texture analysis for differentiating UEC from UCS, we established that the T2WI-TFs model demonstrated high sensitivity and accuracy. The model presented a substantial AUC value of 0.878, potentially attributing to the ability of texture analysis to offer quantitative insights into properties such as roughness, uniformity, coarseness, and patterns within selected ROIs, properties not discernible by the naked eye to distinguish between UEC and UCS. Furthermore, the combined model using one cMRFM and two MRI-T2WIs exhibited the highest AUC value of 0.915 among the four models compared, which was significantly higher than that of the cMRFM model, T2WI-TFs model, and the ADC-TFs. These findings suggest that the relatively limited capability of texture analysis, when focused solely on the largest cross-section in T2-weighted sagittal images, may be effectively complemented by evaluating the areas of early enhancement throughout the tumor using cMRFM.

The present study demonstrated significant distinctions in the differentiation between UEC and UCS, like Kamishima's study findings in univariate analysis of seven items: solid components intensity on T2WI, predominant signal intensity (SI) on T2WI, markedly hyperintense area on T2WI, markedly hypointense area on T2WI, the presence of hyperintense areas in tumors on T1WI, percentage of intertumoral contrast defect area volume, and degree of tumor enhancement [10]. The markedly hypointense area on T2WI and the percentage of the volume of the contrast defect area were also selected for the MRFM model. These findings indicate the presence of necrosis and hemorrhage, which are characteristic of UCS. In addition, the present study identified significant differences regarding early enhancement areas, which were not considered in the study by Kamishima et al. and were selected for both the cMRFM and combined models. Since previous reports have shown that type II endometrial carcinomas show contrast enhancement on dynamic MRI compared to type I carcinomas [23], this result may be due to the higher proportion of type II cancer cells in the UCS, excluding the sarcoma component.

In a previous study by Genever et al. [12], it was reported that the ET/AP ratio was useful in distinguishing between UCS and UEC. However, the findings of the present study did not show a significant difference in this regard. Several potential explanations for these contrasting results can be considered. Uterine leiomyoma is a prevalent benign gynecological disorder among women in their 40s, and it is worth noting that the average volume of leiomyomas in East Asian women exceeds that in Caucasian women by approximately 67% [24]. Therefore, the size of the myomas could have influenced the measurements of the ET/AP ratio. Additionally, it is important to acknowledge that the average age of the patients enrolled in this study was 60 years, which is higher than the age range typically observed in previous reports. As a result, it is possible that the atrophy of the normal uterine muscle may have been more pronounced compared to the atrophy of the uterine leiomyomas, thus affecting the ET/AP ratio.

ADC values reflect tissue density and have been compared between UCS and UEC in various MRI studies. Takeuchi et al. [11] found no significant difference in mean ADC values between the two, but in our study, significantly higher ADC values were observed in UCS, similar to Takahashi et al. study results [25]. This difference may have resulted from differences in case composition and measurement methods. In our study, there were more cases of high-grade UECs, such as G2 and G3. In addition, the entire tumor, including necrosis and cysts, was evaluated. From these points of view, ADC values may have been more discriminative. It was expected that the ADC-TFs model would be more discriminative than the cMRFM or T2WI models, but the results contradicted this expectation: the ADC-TFs model had a lower AUC than the T2WI-TFs model, and the combined model did not incorporate ADC-based TFs. This unexpected result may be due to the increased sensitivity of texture analysis to technical nuances such as variations in diffusion gradient strength and orientation, image noise, and motion artifacts. In support of this, half of the TFs selected for both the T2WI and ADC maps were first-order histogram-based features known to be robust to noise [26].

On the combined model, the first-order median and GLCM sum average were selected for the T2WI-TFs. In terms of the first-order median, UCS produced higher values than UEC. The first-order median values can be used to describe the overall brightness of an image, where high values indicate brightness and low values indicate darkness [27]. This result supports the observation that a high proportion of prominentSI on T2WI and markedly hyperintense areas on T2WI of cMRFM were also confirmed in the texture analysis. Similarly, the GLCM sum averages on T2WI of UCS were higher than those of UEC. GLCM, which is a matrix that represents the combined discretized intensities (gray levels) of adjacent pixels or voxels, measures the relationship between the occurrence of pairs with lower and higher intensity values [28].

Our study had several limitations. First, it was a retrospective study conducted in a small population. Therefore, prospective studies with larger populations are needed to validate and confirm our findings. Second, the texture analysis in this study included the entire tumor ROI, which may have included necrosis or hemorrhage within the tumor. However, previous studies on texture analysis using malignant tumors have been conducted in a similar manner because this method could reflect the nature of the entire tumor and allow for fewer interobserver differences [29]. Third, despite the increasing number of reports on texture analysis using three-dimensional (3D) ROIs, we conducted our texture analysis using two-dimensional (2D) ROIs on the largest tumor slices. This decision was based on the fact that several reports have demonstrated that the performance of texture analysis using 2D ROIs was equal to or better than that of 3D ROIs [30]. Furthermore, using 2D ROIs offers several advantages, including easier acquisition, less labor-intensive procedures, lower complexity, and faster processing. Fourth, our study did not incorporate delayed contrast-enhanced MRI in the assessment of texture analysis. This was because the timing of image acquisition during the delayed phase had varied among the cases. Fifth, in this study, we prioritized the reduction of partial volume effects in texture analysis by targeting uterine tumors with a maximum short diameter of 15 mm. However, it is often challenging to differentiate small tumors of UEC and UCS through MRFMs. In the future, we believe it is necessary to conduct studies specifically focusing on smaller tumors. Finally, although some studies have reported the use of automated ROIs, we chose to use manual ROIs. This approach has several advantages. Manual ROIs allow the evaluator to focus on specific parts of the data and exclude unwanted noise and artifacts. They also enable the use of subjective judgment to identify important areas that might be overlooked by automated methods. Furthermore, automated ROI selection algorithms may not always follow standardized protocols, which could impact consistency across different data sets and studies.

## Conclusions

Our study investigated the differentiation between UEC and UCS using conventional MRI findings, measurements, and texture features. The T2WI-TFs model demonstrated high sensitivity and accuracy, providing a novel approach for distinguishing between UEC and UCS. The combined model, incorporating one conventional MRI finding and two T2WI-TFs, exhibited the highest AUC among the models, emphasizing the complementary nature of texture analysis and conventional MRI. The findings suggest the potential clinical utility of these models in improving the preoperative diagnosis of uterine malignancies. However, the study's limitations, including its retrospective nature and small sample size, should be considered. Further prospective studies with larger populations are warranted to validate these findings and explore their clinical implications.
